# The Utility of 3D Left Atrial Volume and Mitral Flow Velocities as Guides for Acute Volume Resuscitation

**DOI:** 10.1155/2015/697327

**Published:** 2015-07-07

**Authors:** Claudia M. Santosa, David D. Rose, Neal W. Fleming

**Affiliations:** Department of Anesthesiology and Pain Medicine, UC Davis School of Medicine, 4150 V Street, PSSB Suite 1200, Sacramento, CA 95817, USA

## Abstract

Left ventricular end-diastolic pressure (LVEDP) is the foundation of cardiac function assessment. Because of difficulties and risks associated with its direct measurement, correlates of LVEDP derived by pulmonary artery (PA) catheterization or transesophageal echocardiography (TEE) are commonly adopted. TEE has the advantage of being less invasive; however TEE-based estimation of LVEDP using correlates such as left ventricular end-diastolic volume (LVEDV) has technical difficulties that limit its clinical usefulness. Using intraoperative acute normovolemic hemodilution (ANH) as a controlled hemorrhagic model, we examined various mitral flow parameters and three-dimensional reconstructions of left atrial volume as surrogates of LVEDP. Our results demonstrate that peak E wave velocity and left atrial end-diastolic volume (LAEDV) correlated with known changes in intravascular volume associated with ANH. Although left atrial volumetric analysis was done offline in our study, recent advances in echocardiographic software may allow for continuous display and real-time calculation of LAEDV. Along with the ease and reproducibility of acquiring Doppler images of flow across the mitral valve, these two correlates of LVEDP may justify a more widespread use of TEE to optimize intraoperative fluid management. The clinical applicability of peak E wave velocity and LAEDV still needs to be validated during uncontrolled resuscitation.

## 1. Introduction

Blood flow across the mitral valve reflects the instantaneous pressure difference between the left atrium and left ventricle. The pattern of flow is modified by left ventricular diastolic function [1]. Consequently, following the initial descriptions [2], two clinical applications of mitral flow measurements have been investigated: noninvasive calculations of intracardiac pressures [3, 4] and characterization of left ventricular diastolic function [5, 6]. Although the majority of the published literature has focused on these pressure and function assessments as monitors and guides for chronic therapy or as predictors of long term outcomes, they may also have utility during acute patient management, guiding optimization of intravascular volume [7, 8].

Transthoracic and transesophageal echocardiography (TEE) are used with increasing frequency to evaluate and monitor cardiac function in the perioperative setting. The diagnosis and management of the patient with hemodynamic instability are a level 1 indication for intraoperative TEE [9]. In addition, there is growing evidence that optimization of intraoperative cardiac function is associated with a decreased incidence of postoperative complications and shorter lengths of hospital stay [10]. The foundation of cardiac function assessment is left ventricular end-diastolic pressure (LVEDP) measurement. However, because of difficulties and risks associated with direct measurement of LVEDP, many correlates are used, ranging from pulmonary arterial catheter pressure measurements to less invasive TEE-based calculations. Mitral flow measurements form the basis of these TEE assessments. Additional measurements of tissue Doppler [11], pulmonary venous flow [12], color m-mode Doppler [1], or left atrial volume [13] can be incorporated to improve the accuracy of pressure calculations or left ventricular functional characterizations [14], but this increasing complexity may decrease its clinical utility, especially in the acute care setting.

For acute patient management, the most common TEE characterization of ventricular function is the left ventricular end-diastolic volume (LVEDV) [9, 15]. Although it has been shown to be quite sensitive to changes of intravascular volume [16], its use in this setting is limited by difficulties obtaining the most commonly used transgastric short axis image, the potential for regional wall motion abnormalities (RWMA) to produce misleading measurements, and the length of time required to measure LVEDV. In contrast, pulsed wave Doppler assessment of mitral flow is an image that can be obtained reliably and is amenable to rapid quantitative measurements [17]. Also, in contrast to left ventricular assessments, left atrial images are easily and reliably obtained, dynamic changes are not skewed by RWMA, and area measurements can be rapidly obtained using automated border detection algorithms. The potential applications of these additional echocardiographic measurements to guide acute resuscitation or volume optimization have not been extensively evaluated [8]. Using intraoperative acute normovolemic hemodilution (ANH) as a controlled hemorrhagic model we explored the potential utility of mitral flow and left atrial volume as markers of known changes in intravascular volume.

## 2. Methods

These data were collected as part of a single center, prospective, nonrandomized, observational study recruiting from consecutive patients scheduled for elective open prostatectomy, cystectomy, cystoprostatectomy, or anterior/posterior spinal fusion procedures that included ANH as a routine part of the perioperative strategy to minimize the need for homologous blood transfusion. Other results from this protocol have been previously reported [18].

After approval by the University of California Davis Human Subjects Research Committee, all patients provided written, informed consent prior to enrollment. Patients were excluded from consideration if there were contraindications to ANH or esophageal pathology that increased the risks associated with the placement of an ultrasound probe. All patients received preoperative intravenous sedative (midazolam 0.01–0.02 mg/kg). Upon arrival in the operating room, they were preoxygenated and standard physiological monitors were applied. All urological surgical patients received a single intrathecal injection of morphine (0.3 mg). Intravenous fentanyl (1–3 mcg/kg) and propofol (2-3 mg/kg) were then administered to induce general anesthesia. Neuromuscular blockade was established with rocuronium (1 mg/kg) to facilitate endotracheal intubation and provide muscle relaxation during surgery. General anesthesia was maintained with sevoflurane in an air/oxygen mixture to achieve a fractional inspired oxygen concentration of 0.6. Normocapnia was maintained with tidal volumes averaging 8 mL/kg and a positive end-expiratory pressure of 5 cm H_2_O. A radial arterial catheter was then placed, large bore peripheral venous access was established, and the stomach was suctioned with an 18Fr orogastric tube to enhance image acquisition prior to insertion of the TEE probe (V5M probe, Siemens Sequoia Cardiovascular System Model #C512, Siemens Medical Solutions, Malvern, PA).

After a stable anesthetic state had been established, baseline hemodynamic parameters were recorded along with the TEE images described below. The acute normovolemic hemodilution protocol was then initiated. Total blood volume was estimated using the standard formula of 70 mL/kg for male and 65 mL/kg for female patients. Blood removal was accomplished via the peripheral venous catheter with the use of a tourniquet or via the arterial line. 15% of the estimated blood volume (EBV) was removed in three separate aliquots, each one 5% of the EBV. Each aliquot was withdrawn over approximately 10 minutes, during which time the patient's vital signs were continuously monitored to ensure tolerance to this controlled hemorrhage. Blood was collected in standard citrate phosphate dextrose solution blood packs (Baxter Healthcare Corp., Deerfield, IL), agitated to prevent clotting, marked with the patient's information, and kept in the operating room at room temperature until it was reinfused after complete surgical hemostasis. After a total of 15% of the EBV had been removed, the intravascular volume was then replaced, also in three 5% of the EBV aliquots, using an equal volume of 6% hetastarch in lactated electrolyte solution (Hextend, Hospira, Inc., Lake Forest, IL). All hemodynamic measurements and TEE recordings were repeated after each 5% EBV aliquot removal and replacement. The time to complete the ANH and data collection averaged approximately 1 hour. Seven study points were thus defined by this protocol (baseline, EBV −5%, −10%, and −15%, hemodilution to −10% EBV, −5% EBV, and original EBV). If a patient became hemodynamically unstable at any time during the blood removal or replacement (greater than 15% drop in the mean arterial pressure (MAP)) a phenylephrine bolus of 0.1 mg was given. If the hypotension did not resolve, a phenylephrine infusion was started and titrated to maintain the MAP ≥ 50 mmHg. The infusion was tapered and stopped when the MAP was ≥60 mmHg. Physiologic measurements were recorded at least five minutes after a phenylephrine bolus or change in the infusion rate. Two anesthesia care teams were involved in the study; one was solely focused on the patient's care and the other was responsible for data collection.

For each study point defined above, the long axis view of the left ventricle was obtained with the TEE probe positioned in the mid-esophageal four-chamber (ME 4CH) view. Care was taken to optimize LV chamber size and avoid foreshortening. The probe was then rotated to position the left atria and ventricle in the center of the imaging plane. Three-dimensional software was then used to acquire a series of EKG-gated images spanning a 180° rotation of the transducer at 5° intervals (total of 36 images). These images were then stored for offline 3D reconstruction and assessment of left atrium end-diastolic volume, end systolic volume, and ejection fraction using proprietary software* four*Sight (Research Arena, TomTec Imaging Systems, Unterschleissheim, Germany). For these volumetric measurements, the endocardial border was traced in four equally spaced orthogonal pairs of images selected from the 3D reconstruction, that is, each one twenty-two and one-half degrees from the previous measurement. TEE image acquisition was supervised by an anesthesiologist board certified in advanced perioperative TEE and, for consistency, all of the left atrial volumetric analyses were performed by one researcher.

With the TEE probe in the same ME 4CH position, color flow Doppler imaging was used to detect any significant mitral valvular disease. Transmitral flow was then assessed using pulsed wave Doppler with the sample window placed at the tip of the mitral leaflets. After the transmitral flow image was optimized, a still image store was recorded for subsequent measurements of peak E wave amplitude, peak A wave amplitude, E/A ratio, E wave deceleration time, A wave deceleration time, and velocity-time integrals (VTI) for both waves.

Unless noted, all values are presented as mean ± SD. For measurements repeated over time, a one-way ANOVA was used with Dunnett's multiple comparison test to determine statistically significant differences compared to baseline across the seven conditions of graded blood removal and replacement (GraphPad Prism, ver. 6.05, GraphPad Software, San Diego, CA). For all comparisons, a *P* value of <0.05 was considered statistically significant.

## 3. Results

A total of 48 patients were consented for this protocol, all of whom were either ASA I or ASA II. No one was eliminated from the study due to intolerance of the hemodilution. However, a total of seven patients were dropped from analysis: two due to a change in the surgical procedure, one due to the loss of venous access, and four due to the TEE machine not being available. Of the remaining 41 patients, 36 had images of sufficient quality for volumetric analysis. This group included 31 males and 5 females. Their average age was 59 ± 6.4 years, weight 90 ± 14.4 kg, height 174 ± 9.5 cm, and BMI 29 ± 4.4. Surgical procedures included 26 prostatectomies, 4 cystectomies or cystoprostatectomies, and 6 anterior/posterior spinal fusions. Mitral flow data was attempted in 32 patients. All of these patients had images of adequate quality for analysis. This group included 26 males and 6 females. Their average age was 58 ± 6.6 years, weight 85 ± 13.4 kg, height 174 ± 8.8 cm, and BMI 28 ± 4.5. Surgical procedures included 26 prostatectomies, 5 cystectomies or cystoprostatectomies, and 5 anterior/posterior spinal fusions.

**Figure 1:**
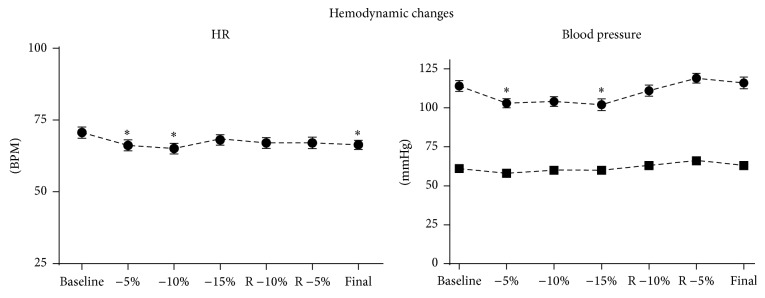
Hemodynamic changes; “*∗*” indicates *P* < 0.05 versus baseline.

**Figure 2:**
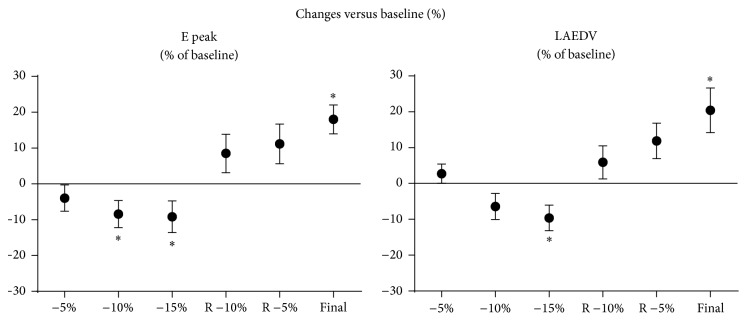
Changes versus baseline (%); “*∗*” indicates *P* < 0.05 versus baseline, E Peak: peak E wave velocity, LAEDV: left atrial end-diastolic volume.

No clinically significant changes in heart rate or blood pressure were observed during the ANH protocol (Figure 1). The measurements of left atrial volume and mitral flow parameters during each of the seven measurement points are collated in Table 1. Only the LAEDV and peak E wave velocity demonstrated statistically significant changes that correlated with the known changes in intravascular volume associated with ANH. At the −15% of EBV time point the LAEDV was −10 ± 21.4% of the baseline value while the peak E wave velocity was −9 ± 25.4% of baseline (Figure 2). Similar changes were also seen at the final study point when the EBV had been restored with Hextend. LAEDV actually overcorrected to 20 ± 37.5% greater than baseline and the peak E wave velocity similarly increased to 19 ± 22.8% greater than baseline.

## 4. Discussion

Acute volume resuscitation and optimization of intracardiac pressures (preload) are a dynamic situation best guided by continuous monitors of cardiac function. The pulmonary arterial (PA) catheter has historically been considered the gold standard in this setting, but it is used with decreasing frequency due to the limits of the information it provides and the risks and complications associated with its placement and use [19]. Central venous pressure monitoring is sometimes used as an alternative, but its placement is associated with similar risks and complications and the clinical utility of the information provided is limited, at best [20]. Transesophageal echocardiography presents an alternative continuous, real-time monitoring option for cardiac function. When compared to the pulmonary arterial catheter it is less invasive with fewer associated risks and complications.

In the clinical setting of acute volume resuscitation, the goal is to optimize the preload of the left ventricle (LVEDP) to maximize cardiac function as guided by the Frank-Starling relationship between LVEDP and cardiac output. The PA catheter uses PCWP as a surrogate for LVEDP, but correlations may be limited by left atrial or mitral valvular pathology. In contrast, the TEE uses the left ventricular end-diastolic volume (LVEDV) as a more direct surrogate for LVEDP and is now a commonly used monitor for this application [9]. It has been demonstrated to be a sensitive measure, responding to changes as small as 2.5% of the intravascular volume [16], and the accumulated data in support of its use makes it one of the few level 1 indications for intraoperative TEE [9]. However, there are limits. Image acquisition can sometimes be difficult. If a transgastric sort axis view is used, RWMA can lead to misleading assessments. More comprehensive characterizations can be obtained using orthogonal mid-esophageal LV long axis views, but for these, as well as the transgastric short axis images, quantification of LVEDV can be tedious, leaving subjective assessments as the most common guide, despite the critical nature of this clinical setting. A monitor that could provide more easily obtained quantitative measurements would be beneficial and decrease the requirements for clinical experience in the assessment of clinical changes during this dynamic period.

TEE can also be used to indirectly calculate the LVEDP. Shortly following the initial description of Doppler measurement of mitral flows by Kitabatake et al. [2], the potential use of these values to calculate LVEDP was demonstrated. Subsequent studies have shown improved accuracy of these calculations when tissue Doppler [11], pulmonary venous flow [12], color m-mode Doppler [1], and 2D left atrial volume [13] measurements are also considered [1]. Unfortunately, as a result, the calculations become too complex to be of much use in the acute clinical setting [1, 12, 13]. The majority of investigations of mitral flow patterns and left atrial volume measurements have focused on using this information to characterize the left ventricular diastolic function as a predictor of long term outcomes or monitor of chronic heart failure therapies [5, 6].

The results of this study demonstrate the potential utility of mitral flow patterns as guides in the acute care setting. In contrast to the transgastric short axis view of the left ventricle, Doppler flow images across the mitral valve are easily and reliably obtained even in critically ill patients [17]. The measurement point at the tip of the mitral leaflets is standardized and reliably reproduced and the flow patterns are easily quantitated. Our results support a single measurement of the peak E wave velocity for this application. Furthermore, just as measurements of the left atrial volume can increase the predictive value of mitral flow patterns regarding long term outcomes, our results indicate that acute changes in left atrial volume may also be used to guide acute resuscitation. Left atrial images are similarly easy to obtain and the more distinct tissue boundaries make them easily quantified.

This study has some limitations that should be highlighted. First, these left atrial volume calculations were done offline using very early versions of the analytical software. Increasing computing capabilities have supported the evolution of TEE imaging options. The initial characterizations of left atrial volume applied Simpson's method of discs to biplanar images, despite the known inaccurate geometric assumptions. Three-dimensional imaging has become routine and provides particular advantages when applied to the irregular shape of the left atrium. Similarly, advances in echocardiographic edge detection software [21] combined with programs designed specifically for the left atrium [22] have been shown to correlate well with other volumetric imaging modalities and make a continuous display of left atrial volume possible. The utility of this more current software should be evaluated with respect to this and similar clinical applications. A second limitation to be considered is the potential for incomplete visualization of the left atrium because of the smaller offset between the esophagus and the left atrium. There are often shoulder areas that extend beyond even the widest possible sector scanning angles. Without the use of a device that could manipulate this offset distance, it is not possible to calculate exactly how much of the left atrium is not visualized and measured; however, our results suggest that this is a relatively small percentage of the total atrial volume. Furthermore, when used in a clinical setting, comparisons would necessarily be made with the baseline values for that patient rather than to a standard “normal” value, further decreasing the impact of any incomplete volume measurements. The potential advantages of this measurement still appear to outweigh the disadvantages associated with left ventricular measurements. A final limitation that should be highlighted is that despite the substantial changes in intravascular volume associated with this ANH protocol, they occurred in a controlled and predictable fashion. A prospective evaluation of these findings in a less controlled resuscitation combined with assessments of cardiac function and clinical outcomes is still required.

## 5. Conclusions

The use of ANH as a controlled human hemorrhagic model allowed the demonstration of the potential utility of 3D left atrial volume measurements and peak E wave mitral flow velocity as echocardiographic monitors that may be used as guides for acute volume resuscitation. Further evaluation in other clinical scenarios is merited.

## Figures and Tables

**Table 1:** Average values for left atrial volume and mitral flow measurements.

Study point	Baseline	EBV-5%	EBV-10%	EBV-15%	Hemodilution to −10%	Hemodilution to −5%	Restored EBV
LAEDV (cm^3^)	47 ± 13.7	48 ± 15.5	**44 ± 16.6** ^*^	43 ± 15.4	49 ± 17.0	52 ± 15.3	**55 ± 19.2** ^*^
Peak E (s)	0.61 ± 0.146	**0.57 ± 0.142** ^*^	**0.54 ± 0.115** ^*^	0.53 ± 0.155	0.64 ± 0.153	0.66 ± 0.185	**0.72 ± 0.156** ^*^
Peak A (s)	0.48 ± 0.163	0.43 ± 0.138	0.42 ± 0.146	0.44 ± 0.148	0.47 ± 0.138	0.47 ± 0.141	0.53 ± 0.191
E/A ratio	1.3 ± 0.33	1.4 ± 0.39	1.4 ± 0.44	1.3 ± 0.44	1.3 ± 0.38	1.4 ± 0.38	1.4 ± 0.43
E decel (ms)	152 ± 60.9	165 ± 60.2	180 ± 72.0	150 ± 58.8	167 ± 60.6	168 ± 50.9	160 ± 50.8
A decel (ms)	114 ± 46.6	124 ± 51.5	114 ± 46.4	113 ± 38.5	133 ± 60.0	120 ± 49.0	115 ± 31.3
E VTI (cm)	0.12 ± 0.106	0.10 ± 0.035	0.11 ± 0.091	0.11 ± 0.129	0.11 ± 0.034	0.12 ± 0.035	0.12 ± 0.038
A VTI (cm)	0.06 ± 0.026	0.06 ± 0.028	0.08 ± 0.126	0.06 ± 0.026	0.07 ± 0.041	0.07 ± 0.039	0.07 ± 0.030

All values are mean ± SD. ^*^ *P* < 0.05 versus baseline. LAEDV: left atrial end-diastolic volume, Peak E: peak E wave velocity, Peak A: peak A wave velocity, E decel: E wave deceleration time, A decel: A wave deceleration time, E VTI: E wave velocity time integral, and A VTI: A wave velocity time integral.
